# Management of and risk factors for regional recurrence in upper lip squamous cell carcinoma: Erratum

**DOI:** 10.1097/MD.0000000000010243

**Published:** 2018-03-16

In the article, “Management of and risk factors for regional recurrence in upper lip squamous cell carcinoma”^[1]^, which appeared in Volume 96, Issue 45 of *Medicine*, Fei Liu's affiliation should be Department of Stomotalogy, the First Affiliated Hospital of Zhengzhou University, Zhengzhou, P.R. China.
